# The molecular profile of gastric intraepithelial foveolar type neoplasia based on somatic copy number alterations and multiple mutation analysis

**DOI:** 10.1007/s10120-024-01543-0

**Published:** 2024-08-12

**Authors:** Tamotsu Sugai, Noriyuki Uesugi, Mitsumasa Osakabe, Ryuya Yamamoto, Koichi Hamada, Michitaka Honda, Naoki Yanagawa, Hiromu Suzuki

**Affiliations:** 1 Department of Molecular Diagnostic Pathology, School of Medicine, Iwate Medical University, 2-1-1, Shiwagun’yahabachou, 028-3695 Japan; 2 Diagnostic Pathology Center, Southern Tohoku General Hospital, 7-115, Yatsuyamada, Kooriyama City, Fukushima 963-8563 Japan; 3 Department of Minimally Invasive Surgical and Medical Oncology, Fukushima Medical University, 1 Hikarigaoka, Fukushima, Fukushima 960-1295 Japan; 4 Department of Surgery, Southern-Tohoku General Hospital, 7-115, Yatsuyamada, Koriyama City, Fukushima 963-8563 Japan; 5 Department of Molecular Biology, School of Medicine, Sapporo Medical University, Sapporo, Japan

**Keywords:** Foveolar type neoplasia, Gastric cancer, Molecular profile, Mutation, Somatic copy number alteration

## Abstract

**Background:**

Gastric foveolar type neoplasia is a rare histological variant of gastric tumors. It is very difficult to differentiate between benign and malignant intraepithelial foveolar neoplasia (IFN). Although limited molecular alterations have been identified in IFNs, somatic copy number alterations (SCNAs), which are linked to tumor progression, have not been systematically evaluated in IFN.

**Methods:**

The aim of the present study was to comprehensively examine SCNAs using a SNP array in 37 cases of IFN, compared with intestinal type dysplasia, including 39 low grade (LGD) and 32 high grade dysplasia (HGD) cases. In addition, gene mutations were evaluated using a gene panel. Finally, we attempted to determine molecular profiles using a hierarchical clustering analysis.

**Results:**

Two patterns could be categorized according to the SCNAs in 108 tumors examined: high (subgroup 1) and low (subgroup 2) frequencies of SCNAs. Although IFN and LGD were associated with subgroup 2, HGD was found in both subgroups. The median numbers of total SCNAs and copy number gains were higher in IFN or HGD than in LGD. In addition, the IFN genotype was characterized by altered genes located at 4p13–4q35.2, including *RAP1GDS1* and *LEF1*, which may be associated with IFN development. Finally, no significant mutations were found in IFNs using a gene panel.

**Conclusions:**

The current molecular profiles of IFN may help elucidate the mechanisms of IFN development.

**Supplementary Information:**

The online version contains supplementary material available at 10.1007/s10120-024-01543-0.

## Introduction

Gastric epithelial neoplasia (dysplasia) is classified morphologically into intestinal and gastric phenotypes [1]. Although limited genetic alterations distinguishing these subtypes have been reported, significant clinicopathological and molecular differences exist between them [2]. The intestinal type phenotype (ID) is frequently encountered in routine practice [3, 4], and its carcinogenesis has been well investigated [2–9]. However, detailed molecular alterations of the gastric phenotype remain unknown.

The gastric type phenotype is sub-classified into foveolar, pyloric, and fundic types by the WHO [2, 10]. Among these, foveolar type neoplasia (FTN) is a rare histological variant of gastric cancer (GC) [2, 10], with histological similarities to foveolar epithelium, including low nuclear pleomorphism [2], low nuclear/cytoplasm ratio (N/C) ratio, and hyperchromatin. It is difficult to differentiate benign from malignant intraepithelial FTN (also known as foveolar type neoplasia in situ [IFN]), even for gastrointestinal pathology experts, due to little nuclear atypia and a low N/C ratio [2]. We reported that genomic changes, such as allelic imbalances (AIs), play important roles in the development of gastric FTN [11, 12]. AIs, thought to be indicators of genetic instability and aggressive nature, are frequently found in malignant versus benign tumors. AIs at 1p, 5q, 18q, and 22q are frequently found in IFN compared with LGD [2], suggesting that despite the low grade nature of the lesion, IFN with AIs at multiple foci may be prone to progression [3, 11]. However, our previous study of AIs was non-comprehensive [3, 4]; therefore, whole genome analyses, such as somatic copy number alterations (SCNAs) closely associated with tumor progression, are necessary to identify comprehensive genomic alterations [4].

Somatic mutation analysis is important for identifying driver mutations closely associated with tumor progression. However, few specific mutations associated with FTN carcinogenesis have been identified [4]. Extensive mutation signatures of FTN may provide novel insight for understanding FTN tumorigenesis. Recently, a gene panel was used to investigate genetic mutations in tumors. While whole genome sequencing may be needed to detect specific mutations in FTN, the limited number of genes included in the gene panel was sufficient to evaluate mutations in tumor cells, given that a limited number of driver genes contribute to GC development. Here, we examined multiple SCNAs and mutations closely associated with gastric carcinogenesis using a SNP array and a next-generation sequencing (NGS) panel to identify the comprehensive molecular features of gastric IFN to further our understanding of this novel GC subtype.

## Materials and methods

### Patients

We enrolled 108 patients with gastric intraepithelial neoplasia (37 with IFN, 39 with intestinal LGD, and 32 with HGD) diagnosed at Iwate Medical University Hospital and related hospitals during 2015–2019. The LGD and HGD cases were included for comparison with IFN. Informed consent was obtained from all patients, and our study was approved by the ethics committee of Iwate Medical University (reference number: MH2022-088).

**Fig.1:**
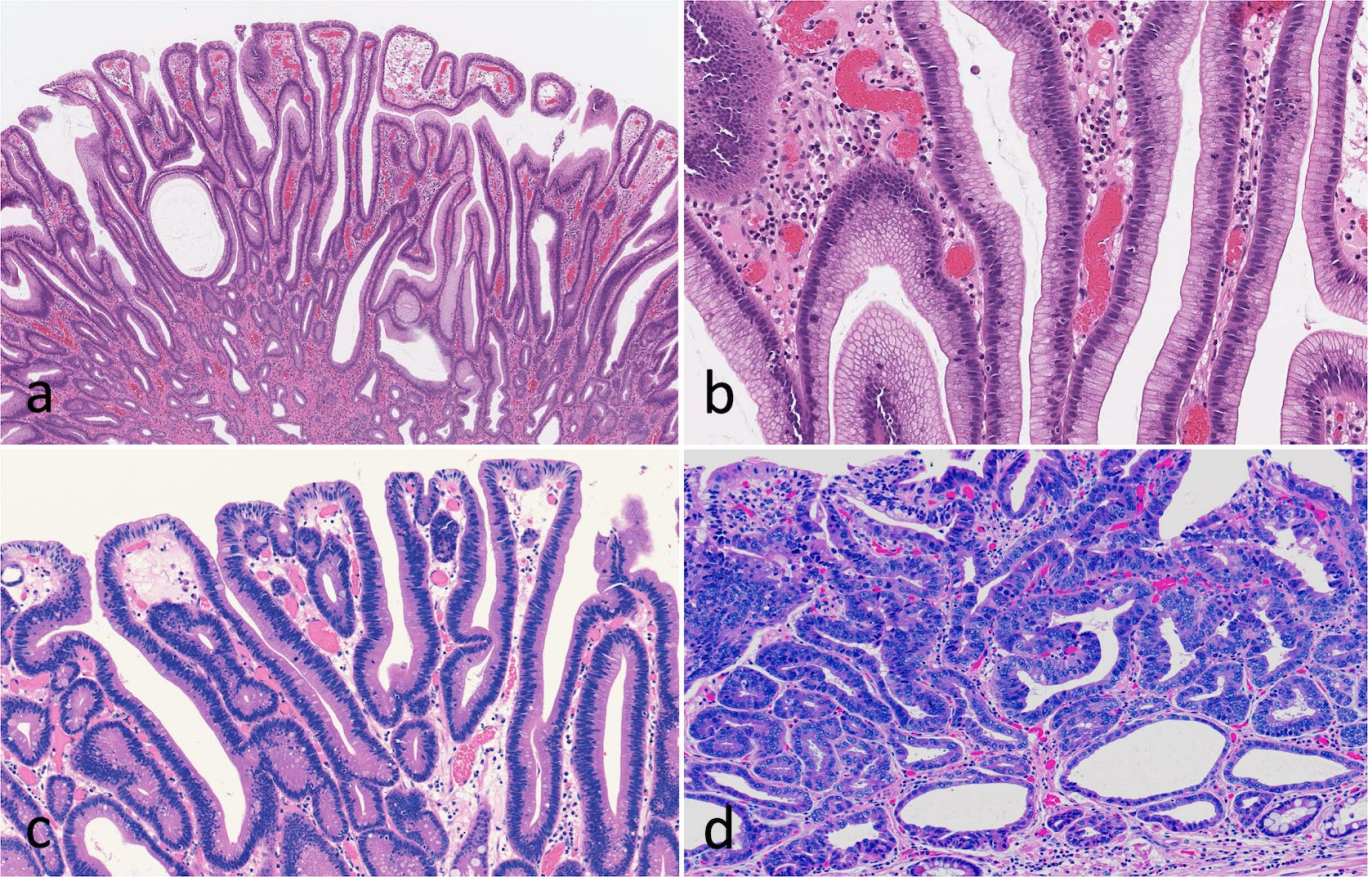
Representative histological findings in gastric intraepithelial neoplasia. **A** Low-magnification view of foveolar type neoplasia. **B** High-magnification view of foveolar type neoplasia. **C** Low grade dysplasia. **D** High grade dysplasia

All tumors were obtained by endoscopic resection. Histopathology reports were available for all patients, and clinicopathological findings including age, sex, lymphovascular invasion status, differentiation type, and tumor invasion depth, were recorded according to the general rules for the management of GC established by the Japanese Gastric Cancer Association [13]. Histological diagnoses were made based on a previous report [2]. Briefly, IFN exhibits cuboidal to columnar cells with pale-to-clear cytoplasm and hyperchromatic round-to-oval nuclei (low N/C). Foveolar-like cells with irregular glandular branching and epithelial folding are also frequently found in IFN, whereas goblet and Paneth cells are rarely observed. In addition, papillary or villous surface structures are obvious in IFN. To confirm the histological diagnosis of IFN, immunohistochemical expression of MUC5AC and MUC6 was assessed. Conversely, LGD resembles colonic adenoma and is composed of large-to-moderate tubules lined by basophilic columnar cells with hyperchromatic pencillate nuclei exhibiting slight pseudostratification and a low N/C ratio. Goblet and Paneth cells are commonly observed in LGD. HGD resembles colonic adenocarcinoma and is composed of irregular glands lined by basophilic columnar cells with hyperchromatic pleomorphic nuclei exhibiting high pseudostratification and a high N/C ratio. Goblet and Paneth cells are rarely found in HGD. The “hybrid type” proposed by Park et al. was not included in this study [14]. Two experienced pathologists (T.S. and N.U.) diagnosed each case by consensus. Finally, IFN we examined was frequently found in severe intestinal metaplasia of the surrounding mucosa. The clinicopathological characteristics of the patients are shown in Table 1, and the representative histological features of the cases are shown in Fig. 1.

**Table 1:** Clinicopathological findings of intraepithelial neoplasia

	Total	IFN (%)	LGD (%)	HGD (%)	*p*-value
Total	108	37 (34.3)	39 (36.1)	32 (29.6)	
Sex					0.0199
Man	85	25 (29.4)^*^	30 (35.3)	30 (35.3)^*^	
Woman	23	12 (52.2)	9 (39.1)	2 (8.7)	
Age, median [range] (year)	71.5 [14–87]	70 [14–85]	73 [48–87]	72 [60–85]	0.5182
Locus					0.7788
U/M/L	20/38/50	8/15/14	6/13/20	6/10/16	
Size, median [range] (mm)	14 [2–84]	11 [2–84]^*^	15 [5–60]	16 [4–60]^*^	0.031
Differentiation					0.0016
Well	99	31 (31.3)	39 (39.4)	29 (29.3)	
Moderately	4	1 (25)	0 (0)	3 (75)	
Papillary type	5	5 (100)	0 (0)	0 (0)	
Mucin type					< 0.0001
Gastric	45	35 (77.8)^†, ‡^	2 (4.4)^†^	8 (17.8)^‡^	
Large intestinal	5	0 (0)	4 (80)	1 (20)	
Small intestinal	23	0 (0)	15 (65.2)	8 (34.8)	
Mixed	35	2 (5.7)	18 (51.4)	15 (42.9)	

*IFN* intraepithelial foveolar neoplasia, *LGD* low grade dysplasia, *HGD* high grade dysplasia, *U* upper portion, *M* middle portion, *L* lower portion

^*^Bonferroni adjusted *p* < 0.05

^†^ and ‡, Bonferroni adjusted *p* < 0.01

### Immunohistochemical analysis

Sections of formalin-fixed, paraffin-embedded tissue blocks were cut at 3–4 μm thickness for immunohistochemical analysis using anti-MUC5AC (CLH2; Novocastra Laboratories), anti-MUC6 (CLH5; Novocastra Laboratories), anti-RAP1GDS1, and anti-LEF1 antibodies. Details of the immunohistochemical method and evaluation are described elsewhere [2, 4].

### DNA extraction

Microdissection of formalin-fixed, paraffin-embedded tumor and non-tumor mucosal sections was performed on hematoxylin-stained slides. The tumor and non-tumor mucosal components were micro-dissected separately according to a previous method [2, 3]. The tumor components were composed of ≥ 50% tumor cells. DNA was extracted from both components by sodium dodecyl sulfate lysis and proteinase K digestion, followed by a phenol–chloroform procedure, as reported previously [2, 4].

### Analysis of microsatellite instability (MSI)

MSI status was determined based on five NCI markers: BAT25, BAT26, D2S123, D5S346, and D17S250. MSI-high status was defined as the presence of two or more unstable markers, MSI-low status as the presence of one unstable marker, and microsatellite stable (MSS) as the absence of instability [15].

### SNP array analysis

SNP array analysis was performed using the OncoScan^™^ CNV Assay (Thermo Fisher Scientific, Inc., UK) as described previously [16]. In brief, the assay involves a microarray consisting of 217,454 molecular inversion probes (MIPs) that bind to target DNA to form an incomplete circular loop, leaving a gap at a specific SNP site. Following annealing, probes are distributed to wells containing either adenosine/thymidine triphosphate or guanosine/cytosine triphosphate nucleotides. Non-circular MIPs and genomic DNA are digested by exonucleases, whereas only closed circular MIPs remain; these MIPs are linearized and amplified. Finally, the resulting fragments are allowed to bind to the OncoScan array and are fluorescently visualized on the GeneChip^™^ Scanner 3000 7G (Thermo Fisher Scientific, Inc.). Fluorescence intensity is correlated with the copy number of the analyzed genomic sites. We used 80 ng DNA per array run. OncoScan raw data files were filtered using the Chromosome Analysis Suite v4.0 (Thermo Fisher Scientific, Inc.). Measurements of individual SNP probes were aggregated to segments of unchanged allele-specific copy numbers using ASCAT v3.1.0 (1).

### Classification of SCNAs

We classified SCNA patterns as gain, loss of heterozygosity (LOH), and copy neutral LOH (CN-LOH). A gain was defined as a gross chromosomal change caused by the gain of the entire gene and surrounding region. LOH was considered a gross chromosomal change resulting in loss of the entire gene and surrounding region. CN-LOH was defined as LOH without a copy number change (CN = 2). Detailed classification criteria are described elsewhere [16].

### NGS

Targeted NGS was performed using micro-dissected specimens. In brief, a NGS library was prepared using a custom panel (Illumina, San Diego, CA, USA), containing 753 amplicons covering 82 exonic regions from 28 genes (*APC*, *BRAF*, *TP53*, *CDKN2A*, *MET*, *ATM*, *MLH-1*, *PMS2*, *HRAS*, *AXIN2*, *BAX*, *DCC*, *MSH2*, *POLE*, *RNF43*, *PTEN*, *EPCAM*, *MSH6*, *BUB1B*, *RHOA*, *KRAS*, *NRAS*, *SMAD4*, *CDK4*, *PIK3CA*, *STK11*, *TGFBR2*, and *EGFR*). Sequencing was achieved for each pool by loading 600 μL of the library. Sequencing analysis viewer software (Illumina) was used to confirm quality metrics with interop files along with run info and parameters. A Phred score of Q30 was considered for each run. MiSeq Reporter software (Illumina) was used for demultiplexing, sequence alignment, and variant calling. A FASTQ file for each sample pool and a single genomic variant call file were generated from successful sequencing runs.

### Determination of pathological mutations

Annotation of detected variants was performed using Illumina Variant Studio v2.2 software (Illumina). Every variant with an allele frequency < 10% was removed before review. Detected variants were marked with a PASS filter flag if the following criteria were met: the variant was present in each pool, the cumulative depth was 1000 × per pool, and the average depth was 500 × per pool. Variants were classified using the ClinVar (http://www.ncbi.nlm.nih.gov/clinvar) and COSMIC (http://cancer.sanger.ac.uk/cosmic) databases. Pathogenic and likely pathogenic variants were reported according to standard guidelines (http://www.ncbi.nlm.nih.gov/clinvar; http://cancer.sanger.ac.uk/cosmic).

### Statistical analysis

Differences in clinicopathologic variables among groups were analyzed by Fisher’s exact test using JMP Pro 16.1 for Windows (SAS, Tokyo, Japan). Differences in age and tumor size distributions were evaluated using the Mann–Whitney *U* test in JMP Pro 16.1. Comparisons among more than two groups were assessed using the Friedman test. If significant differences among multiple groups were found, differences between two groups were evaluated using the Wilcoxon signed rank test with Bonferroni correction.

## Results

### Overall gene copy number alterations

In the overall cohort, the median number of genes with total copy number alterations per patient was 1728, with a median of 1,252.5 genes with a gain (range: 0–40,285), 0 with LOH (range: 0–18,552), and 1.5 with CN-LOH (range: 0–5577). In subgroup 1, the median number of genes with total copy number alterations per patient was 40,277.5, with a median of 40,277.5 genes with a gain (range: 37,735–40,285), 0 with LOH (range: 0–747), and 0 with CN-LOH (range: 0–1699). In subgroup 2, the median number of genes with total copy number alterations per patient was 1662.5, with a median of 1,063 genes with gains (range: 0–15,116), 0 with LOH (range: 0–18,552), and 2 with CN-LOH (range: 0–5577).

### Hierarchical clustering analysis based on gene copy number alterations in IFN, LGD, and HGD cases with the MSS phenotype

**Fig.2:**
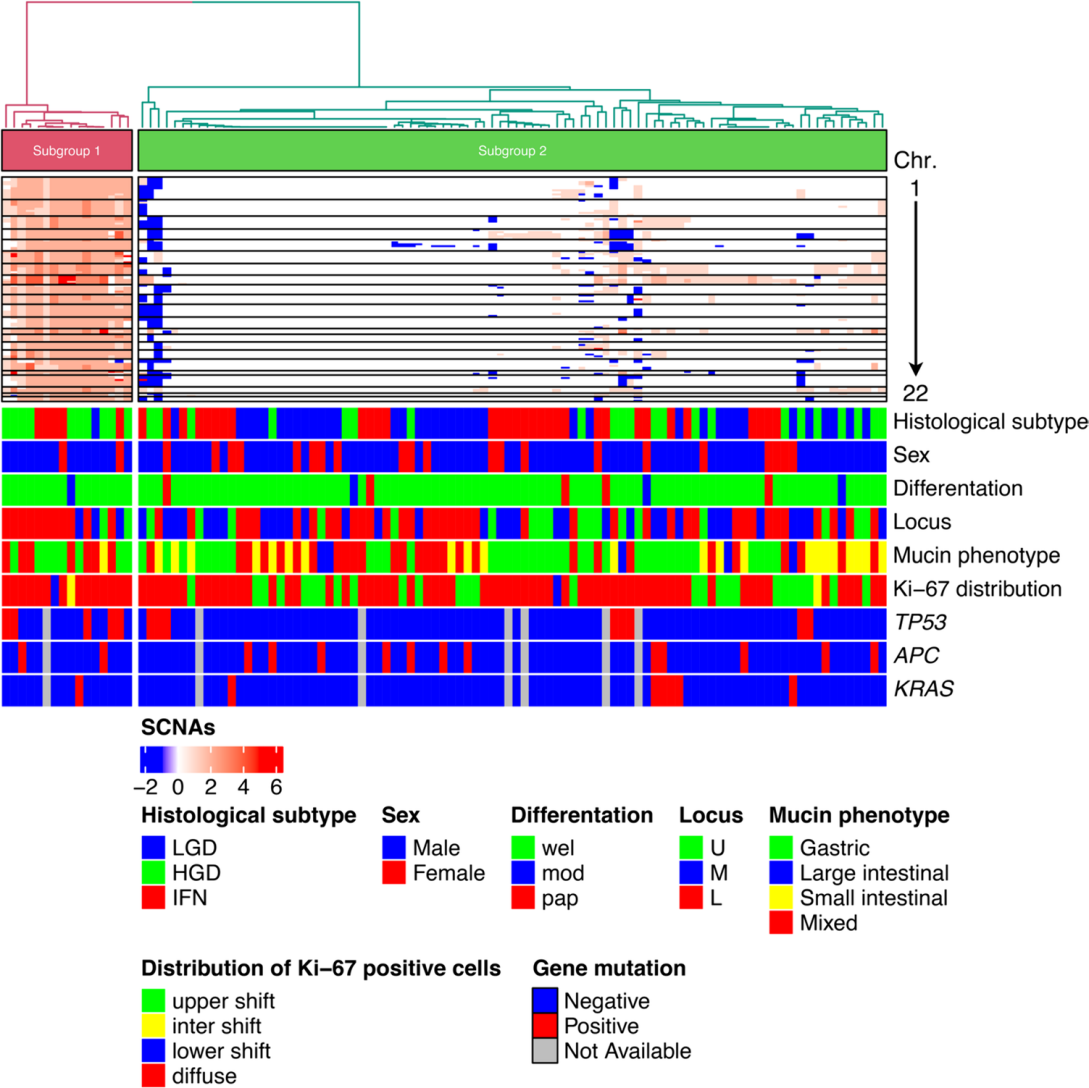
Hierarchical cluster analysis based on somatic copy number alterations in 108 gastric intramucosal neoplasias with the MSS phenotype

We evaluated gene copy number alterations (GCNAs) in lieu of SCNAs to determine copy number changes, given that the number of individual genes mapping to corresponding loci (GCNAs) is easier to calculate than the number of allelic loci (SCNAs). The IFN, LGD, and HGD cases examined were classified as MSS. We performed hierarchical clustering analysis based on the GCNAs, including gains, LOH, and CN-LOH, to examine differences in genetic alterations among the IFN, LGD and HGD cases with the MSS phenotype (Fig. 2).

We classified the IFN, LGD, and HGD cases with the MSS phenotype into two subgroups according to GCNA frequency: high (subgroup 1) and low (subgroup 2) (Fig. 2), and examined the clinicopathological characteristics of each (Supplementary Table 1). The frequencies of IFN and LGD cases were statistically higher in subgroup 2 versus 1. However, no significant difference existed in the frequency of HGD between subgroups. There were no statistical differences in the other clinicopathological findings examined between subgroups.

**Fig.3:**
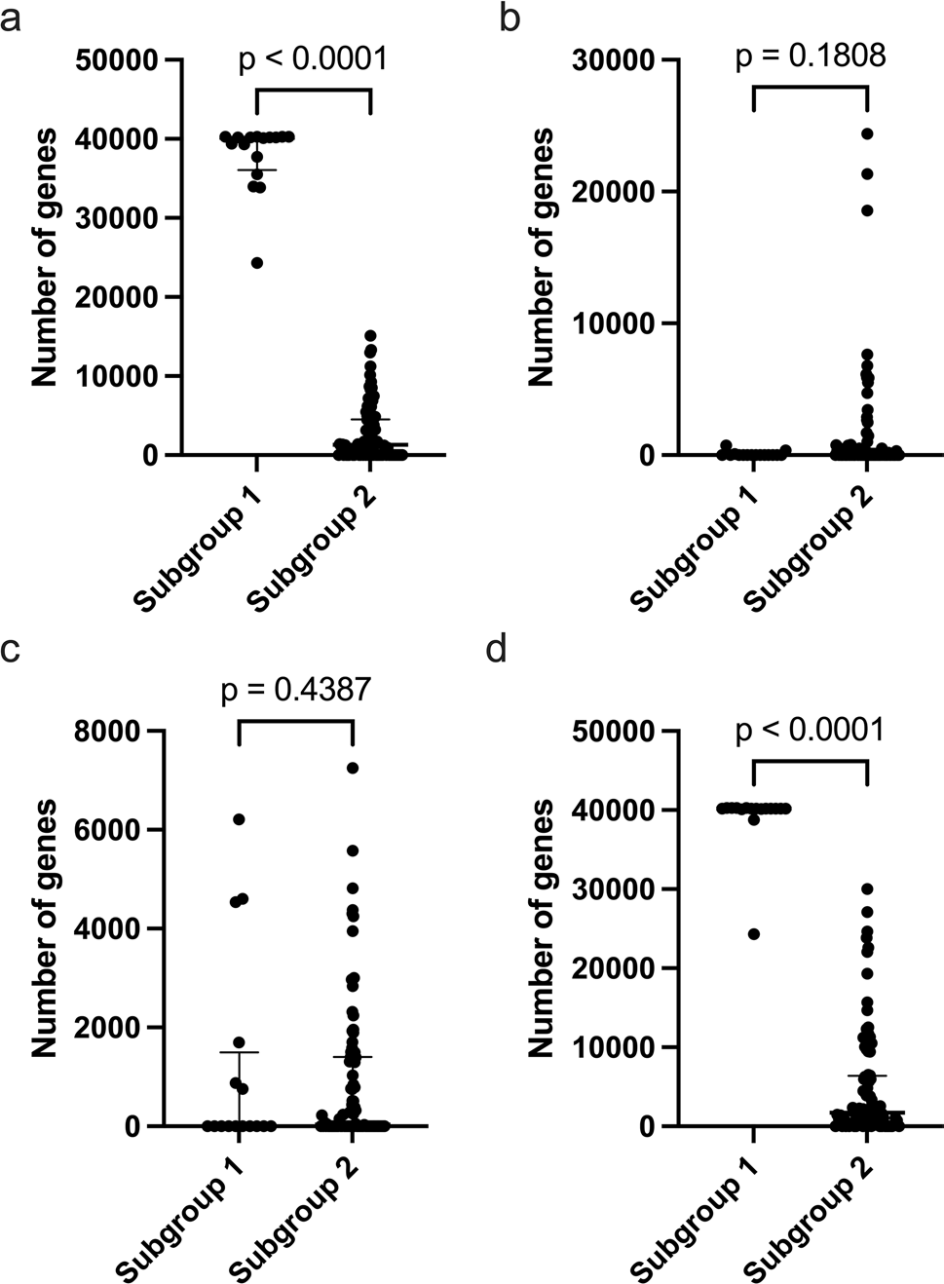
Comparison of the numbers of total GCNAs, gains, copy-neutral LOH, and LOH in 108 gastric intramucosal neoplasia cases with MSS. **a** Comparison of the number of genes with gains between subgroups 1 and 2. **b** Comparison of the number of genes with LOH between subgroups 1 and 2. **c** Comparison of the number of genes with CN-LOH between subgroups 1 and 2. **d** Comparison of the number of genes with total SCNAs between subgroups 1 and 2. Detailed GCNA data for subgroups 1 and 2 are shown

We compared the numbers of genes with gains, LOH, CN-LOH, and total GCNAs between subgroups. Significant differences in the average number of genes with copy number gains were found (*p* < 0.0001) (Fig. 3a). However, the numbers of genes with LOH and CN-LOH were similar between subgroups (Fig. 3b and c). In addition, there was a significant difference in the number of genes with total GCNAs between subgroups 1 and 2 (*p* < 0.0001) (Fig. 3d).

### Somatic gene number alterations in IFN, LGD, and HGD cases with the MSS phenotype

**Fig. 4:**
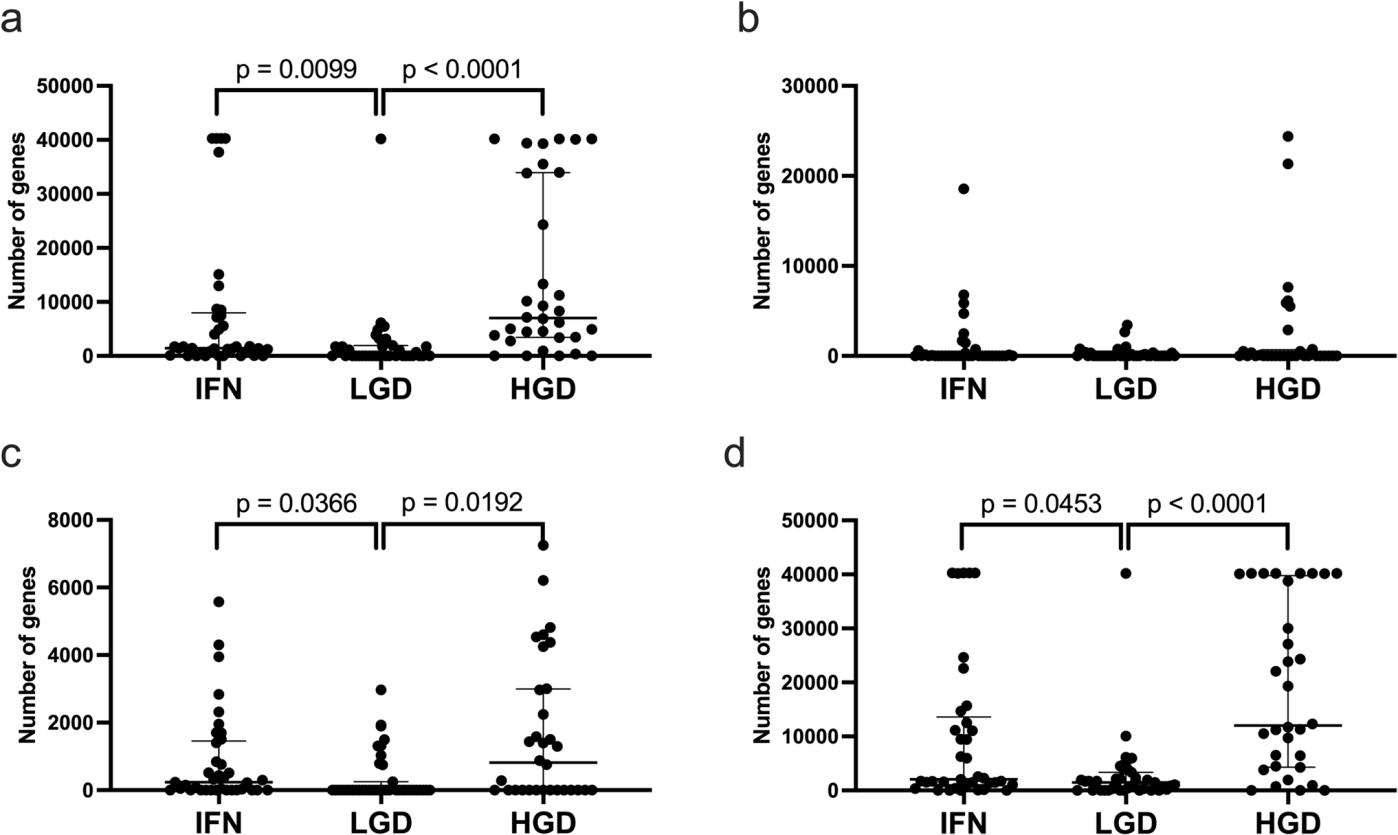
Comparison of the numbers of total GCNAs, gains, CN-LOH, and LOHs in 108 gastric intramucosal neoplasia cases with MSS. **a** Comparison of the total number of genes with gains among the IFN, LGD, and HGD cases. **b** Comparison of the number of genes with LOH among the IFN, LGD, and HGD cases. **c** Comparison of the number of genes with CN-LOH among the IFN, LGD, and HGD cases. **d** Comparison of the number of genes with total SCNAs among the IFN, LGD, and HGD cases. Detailed GCNA data for IFN, LGD and HGD are shown

We compared the numbers of genes with gains, LOH, CN-LOH, and total GCNAs among lesion types (IFN, LGD and HGD). Significant differences in the average number of genes with gains between IFN and HGD or LGD were found (*p* < 0.01; *p* < 0.01) (Fig. 4a). The number of genes with CN-LOH was significantly higher in IFN or HGD than in LGD. However, the number of genes with LOH was similar among lesion types (Fig. 4b and c). Moreover, significant differences existed in the total number of GCNAs among the three lesion types (*p* < 0.01; *p* < 0.001) (Fig. 4d).

Gain events detected in > 30% of cases were located at 3p21.1, 3p14.2–3p14.1, 3p13, 4p13–4q35.2, 8p23.3–8q24.3 in the IFN cases (Supplementary Table 2) versus 1p36.33–1p36.11, 1p35.2–1p31.1, 1p12–1q44, 2p25.3–2q37.3, 3p26.3–3q29, 4p16.3–4p15.32, 4q31.23–4q34.1, 5p15.33–5q11.1, 6p25.3–6q27, 7p22.3–7q36.3, 8p23.3–8q24.3, 9q13–9q34.3, 10p15.3–10q26.3, 11q12.2–11q25, 12p13.33–12p13.32, 12q14.1–12q15, 13q11–13q34, 15q21.3–15q26.3, 16p13.3–16q24.3, 17q11.1–17q25.3, 18p11.32–18p11.21, 18q11.1–18q23, 19q11–19q13.2, 20p13–20q13.33 and 21q21.2–21q22.11 in the HGD cases (Supplementary Table 3). In addition, no LOH or CN-LOH events were detected in more than 30% of cases (Supplementary Table 2, 3).

There was a significant difference in the frequency of gain events (> 30% of cases) between IFN and LGD at 4p13–4q35.2, but no difference (> 30% of cases) in that of LOH or CN-LOH (Supplementary Table 4). In contrast, significant differences existed in the frequencies of multiple gain events (present in > 30% of cases) between IFN and HGD cases, but no such difference in LOH or CN-LOH was observed (Supplementary Table 5). We searched for candidate oncogenes located at 4p13–4q35.2 using the Cancer Gene Census. In this chromosomal region, we identified Rap1 GTPase-GDP dissociation stimulator 1 (*RAP1GDS1*) and lymphoid enhancer binding factor 1 (*LEF1*), which have oncogenic functions. The frequencies of *RAP1GDS1* and *LEF1* mutations were significantly higher in IFN than in LGD (Table 2). There was no significant difference in the frequency of gain events between IFN and HGD.

**Table 2:** Comparison of mutations in 4p13–4q35.2, the locus encompassing *RAP1GDS1* and *LEF1*, between IFN and LGD

Symbol	Location	Function	IFN (%)	LGD (%)	p-value
*RAP1GDS1*	4q23	oncogene, fusion	16 (43.2)	1 (2.6)	0.0020
*LEF1*	4q25	oncogene, TSG	16 (43.2)	1 (2.6)	0.0020

*IFN* intraepithelial foveolar neoplasia, *LGD* low grade dysplasia, *TSG* tumor-suppressor gene

Next, we examined the interaction networks closely associated with RAP1GDS1 and LEF1, respectively, using the STRING database (https://string-db.org). Of the candidate signaling pathways found, Rap1 signaling (potentially downstream of the MAPK and PI3K/AKT pathways) and Wnt signaling were associated with RAP1GDS1 and LEF1, respectively (Supplementary Fig. 1).

### Mutation analysis of IFN, LGD, and HGD using NGS

We examined 28 genes included on a customized gene panel, in which whole exons of *APC* and *TP53* were included, but only hot spots were included for the other 26 genes. The frequency of *TP53* mutation was significantly higher in HGD than in LGD (10 of 32 versus 1 of 39 cases, respectively), but no significant difference existed between IFN and HGD. Although the *APC* mutation frequency was higher in LGD and HGD (7 of 32 and 6 of 39 cases, respectively) than in IFN (1 of 30), the difference was not statistically significant. *APC* nonsense (LGD: 4; HGD: 6) and frameshift (LGD: 2; HGD: 1) mutations were common in LGD and HGD. *TP53* mutations in exons 4–8 were observed. *TP53* and missense and transition mutations were common in each lesion examined. The frequency of *KRAS* mutations was lower in IFN, LGD, and HGD. Detailed mutation results are shown in Supplementary Tables 6–10.

## Discussion

SCNAs are genomic changes intrinsic to disease development [4, 6]. Comprehensive analysis of SCNAs enables identification of potential cancer-induced genomic changes [4, 6]. SCNAs play critical roles in activating oncogenes and inactivating tumor suppressors, and an understanding of the biological and phenotypic effects of SCNAs helps clarify the biological mechanism of neoplastic progression in various cancers [17]. Identifying the SCNAs involved in tumorigenesis enables determination of driver events that contribute to oncogenesis and cancer progression [18, 19]. However, the passenger SCNAs acquired during cancer evolution may not actively contribute to the aggressive nature of cancer cells [6]. Regardless, the accumulation of SCNAs occurs during tumor progression, and positively selected SCNAs tend to recur across cancers at elevated rates [6, 17]. We examined whether SCNAs occur during the early development of IFN, compared with intestinal type LGD and HGD, using high-resolution somatic copy number profiles generated using a single platform. This is the first genome-wide analysis of absolute allelic copy number data in IFN.

In this study, compared with LGD and HGD, IFN was molecularly characterized by multiple GCNAs. This is surprising, since GCNAs are reflected by histological findings including little nuclear atypia and a low N/C ratio [4]. In addition, a previous study showed an epigenotype pattern in IFN different from that in intestinal type GC [2]. Thus, we suggest that gastric intramucosal neoplasia can be largely classified into two molecular phenotypes: genetic- and epigenetic-dominant types [9]. The epigenetic-dominant type is characterized by high DNA methylation and few genomic changes during the early GC development, whereas the genetic-dominant type exhibits accumulated genetic changes (i.e., chromosomal instability) during the early GC. Therefore, IFN is considered the genetic-dominant type, different from the epigenetic-dominant type associated with intestinal type GC. However, the molecular classification of individual gastric adenocarcinomas is not clear-cut. Further investigation is needed to validate this molecular classification. Finally, no consensus exists between Western and Japanese pathologists as to whether IFN is benign or malignant [2, 10]. The present data, however, suggest that IFN acquires malignant characteristics due to the accumulation of multiple SCNAs, indicating an aggressive tumor phenotype [2].

Specific mutations in IFN have not been discovered until now. We examined the mutation status of 28 genes using an NGS gene panel to understand gastric carcinogenesis in IFN. However, no significant mutations in the genes examined were detected in IFN. Whole genome sequencing is necessary to identify significant driver mutations closely associated with IFN development [20, 21]; however, it is costly and time consuming, and thus difficult to conduct [20]. At least, driver mutations that were reported in previous studies were not found using a gene panel that contains driver genes in gastrointestinal cancers. Recent studies have demonstrated that *KLF4* mutation plays an important role in the pathologic characteristics of foveolar-type gastric adenoma in *Helicobacter pylori*-naive patients [22, 23]. Although this is an interesting finding to evaluate the tumorigenesis of IFN, intestinal metaplasia, which may be caused *H. pylori* infection, was frequently found in the surrounding mucosa of IFN in the current cases. This suggests that the clinicopathological and molecular findings we examined may be different from those of *H. pylori*-negative cases. Therefore, we believe that gene mutations play a minor role in IFN pathogenesis with *H. pylori* infections. However, we will examine mutation of the *KLF4* gene in the present IFN cases to identify the mutation profile of IFN.

Using the Cancer Gene Census, we searched for candidate oncogenes associated with IFN pathogenesis located at 4p13–4q35.2, a locus showing a gain in IFN, and identified *RAP1GDS1* and *LEF1*. Two signaling pathways closely associated with RAP1GDS1 (RAP1 signaling) and LEF1 (Wnt signaling) may play a role in IFN development. Rap1 signaling plays a role in many important cellular processes, such as regulation of cell adhesion and cell junctions, cell migration, and cell proliferation and survival [24]. Although the role of *Rap1* signaling in IFN tumorigenesis remains unknown, Wnt signaling may promote IFN development [25]. Candidate signaling pathways specific to IFN development have not been identified, and further functional studies are required to elucidate a signaling pathway closely associated with IFN tumorigenesis.

This study has some limitations. First, the number of IFN cases examined was small because IFN is a rare histological variant of GC [4]. However, we believe that the sample size was adequate to identify molecular alterations in IFN. Second, the invasive phenotype of IFN was not examined, as it is difficult to obtain invasive IFN lesions, even in high-volume gastrointestinal pathology centers. Finally, we could not compare the immunohistochemical expression of RAP1GDS1 and LEF1 between IFN and LGD because reliable and reproducible staining was not obtained from the antibodies used. Development of new antibodies may be necessary to identify differences in the expression of these proteins between the two lesion types. The RNA expression level of both genes can be examined using real-time PCR. However, fresh samples for RNA analysis were not obtained in this study. Moreover, it is difficult to perform quantitative analysis of RNA expression levels consistently using paraffin-embedded tissue.

In conclusion, SCNAs are useful for demonstrating the utility of biomarkers predicting the risk of neoplastic progression. Our study suggests that contrary to expectations, multiple SCNAs accumulate during IFN development. We also showed that IFN is molecularly categorized according to chromosomal instability phenotype. However, specific mutations played a minor role in the development of IFN. In addition, regardless of the scant nuclear atypia and low N/C ratio, we suggest that IFN has a malignant rather than benign nature due to the accumulation of multiple SCNAs. Finally, we suggest that candidate oncogenes associated with IFN pathogenesis are located at 4p13–4q35.2, a locus showing a gain in IFN (*RAP1GDS1* and *LEF1*). Future investigation of the molecular pathogenesis of IFN is required.

## Supplementary Information

Below is the link to the electronic supplementary material.

Supplementary file1 (DOCX 38 KB)

## Data Availability

The data that support the findings of our study are available from the corresponding author upon reasonable request.
