# Supplementary material for: The molecular profile of gastric intraepithelial foveolar type neoplasia based on somatic copy number alterations and multiple mutation analysis
Source: Gastric Cancer. 2024 Aug 12;27(6):1220–8. doi: 10.1007/s10120-024-01543-0 (PMC11513720; doi:10.1007/s10120-024-01543-0)
Supplement: Supplementary file 1 — Supplementary file1 (DOCX 38 KB) [file 10120_2024_1543_MOESM1_ESM.docx]

Supplementary Table 1. Clinicopathological findings in subgroup 1 and 2

|  | Total | Subgroup 1  (%) | | Subgroup 2  (%) | | p-value |
| --- | --- | --- | --- | --- | --- | --- |
| Total | 108 | 16 | (14.8) | 92 | (85.2) |  |
| Sex |  |  |  |  |  | 0.5141 |
| Man | 85 | 14 | (16.5) | 71 | (83.5) |  |
| Woman | 23 | 2 | (8.7) | 21 | (91.3) |  |
| Age, median [range] (year) | 71.5 [14-87] | 70.5 [61-85] | | 71.5 [14-87] | | 0.9758 |
| Locus |  |  |  |  |  | 0.185 |
| U/M/L | 20/38/50 | 2/3/11 | | 18/35/39 | |  |
| Size, median [range] (mm) | 14 [2-84] | 15.5 [4-56] | | 14 [2-84] | | 0.6652 |
| Histological subtype |  |  |  |  |  | 0.0028 |
| IFN | 37 | 5 | (13.5) | 32 | (86.5) |  |
| LGD | 39 | 1 | (2.6) | 38 | (97.4) |  |
| HGD | 32 | 10 | (31.3) | 22 | (68.8) |  |
| Differentiation |  |  |  |  |  | 0.5664 |
| Well | 99 | 15 | (15.2)^*^ | 84 | (84.8)^*^ |  |
| Moderately | 4 | 1 | (25)^†^ | 3 | (75)^†^ |  |
| Papillary type | 5 | 0 | (0) | 5 | (100) |  |
| Mucin type |  |  |  |  |  | 0.3067 |
| Gastric | 45 | 8 | (17.8) | 37 | (82.2) |  |
| Large intestinal | 5 | 0 | (0) | 5 | (100) |  |
| Small intestinal | 23 | 1 | (4.3) | 22 | (95.7) |  |
| Mixed | 35 | 7 | (20) | 28 | (80) |  |

U, upper portion; M, middle portion; L, lower portion; IFN, intraepithelial foveolar neoplasia; LGD, low grade dysplasia; HGD, high grade dysplasia; * and †, Bonferroni adjusted p < 0.01.

Supplementary Table 2: Gain events detected in more than 30% of cases in IFN

| Chromosomal regions | IFN n= 37 (%) | |
| --- | --- | --- |
| **Gain** |  |  |
| 3p21.1 | 12 | (32.4) |
| 3p14.2-3p14.1 | 12 | (32.4) |
| 3p13 | 12 | (32.4) |
| 4p13-4q35.2 | 12-18 | (32.4-48.6) |
| 8p23.3-8q24.3 | 12-17 | (32.4-45.9) |
| **Loss** |  |  |
| None |  |  |
| **CN-LOH** |  |  |
| None |  |  |

IFN, intraepithelial foveolar neoplasia; CN-LOH, copy neutral loss of heterozygosity.

Supplementary Table 3: Gain events detected in more than 30% of cases in HGD.

| Chromosomal regions | HGD n= 32 (%) | |
| --- | --- | --- |
| **Gain** |  |  |
| 1p36.33-1p36.11 | 10 | (31.3) |
| 1p35.2-1p31.1 | 10-11 | (31.3-34.4) |
| 1p12-1q44 | 10-12 | (31.3-37.5) |
| 2p25.3-2q37.3 | 11-13 | (34.4-40.6) |
| 3p26.3-3q29 | 10-14 | (31.3-43.8) |
| 4p16.3-4p15.32 | 10-11 | (31.3-34.4) |
| 4q31.23-4q34.1 | 10 | (31.3) |
| 5p15.33-5q11.1 | 11-16 | (34.4-50) |
| 6p25.3-6q27 | 12-17 | (37.5-53.1) |
| 7p22.3-7q36.3 | 14-21 | (43.8-65.6) |
| 8p23.3-8q24.3 | 16-25 | (50-78.1) |
| 9q13-9q34.3 | 10-11 | (31.3-34.4) |
| 10p15.3-10q26.3 | 11-13 | (34.4-40.6) |
| 11q12.2-11q25 | 10-11 | (31.3-34.4) |
| 12p13.33-12p13.32 | 10 | (31.3) |
| 12q14.1-12q15 | 10 | (31.3) |
| 13q11-13q34 | 15-17 | (46.9-53.1) |
| 15q21.3-15q26.3 | 10-11 | (31.3-34.4) |
| 16p13.3-16q24.3 | 10-13 | (31.3-40.6) |
| 17q11.1-17q25.3 | 10-14 | (31.3-43.8) |
| 18p11.32-18p11.21 | 10 | (31.3) |
| 18q11.1-18q23 | 10-11 | (31.3-34.4) |
| 19q11-19q13.2 | 10 | (31.3) |
| 20p13-20q13.33 | 16-18 | (50-56.3) |
| 21q21.2-21q22.11 | 10 | (31.3) |
| **Loss** |  |  |
| None |  |  |
| **CN-LOH** |  |  |
| None |  |  |

HGD, high grade dysplasia; CN-LOH, copy neutral loss of heterozygosity.

Supplementary Table 4: Significant difference in the allelic locus between IFN and LGD

| Cytoband | IFN (%) | | LGD (%) | | p-value |
| --- | --- | --- | --- | --- | --- |
| **Gain** |  |  |  |  |  |
| 4p13-4q35.2 | 12-18 | (32.4-48.6) | 1-2 | (2.6-5.1) | 0.0007-0.026 |
| **Loss** |  |  |  |  |  |
| None |  |  |  |  |  |
| **CN-LOH** |  |  |  |  |  |
| None |  |  |  |  |  |

Supplementary Table 5 SCNA events detected in more than 30% of cases between IFN and HGD

| Cytoband | IFN (%) | | HGD (%) | | p-value |
| --- | --- | --- | --- | --- | --- |
| **Gain** |  |  |  |  |  |
| 6p21.32-6q13 | 4-6 | (10.8-16.2) | 14-17 | (43.8-53.1) | 0.0469-0.049 |
| 6q24.1 | 5 | (13.5) | 15 | (46.9) | 0.049 |
| 6q24.3-6q27 | 5 | (13.5) | 15 | (46.9) | 0.049 |
| 7p22.1-7p11.2 | 9-10 | (24.3-27) | 19-21 | (59.4-65.6) | 0.0469-0.049 |
| 8q23.3-8q24.3 | 11-15 | (29.7-40.5) | 23-25 | (71.9-78.1) | 0.0469-0.049 |
| 13q11-13q12.3 | 6 | (16.2) | 16 | (50) | 0.049 |
| 13q14.2-13q21.33 | 5-6 | (13.5-16.2) | 16-17 | (50-53.1) | 0.0469-0.049 |
| 13q31.1 | 6 | (16.2) | 16 | (50) | 0.049 |
| 13q31.3 | 6 | (16.2) | 16 | (50) | 0.049 |
| 13q32.3-13q34 | 6 | (16.2) | 16 | (50) | 0.049 |
| 20p13-20p12.1 | 6 | (16.2) | 16-18 | (50-56.3) | 0.0469-0.049 |
| 20p11.21 | 7 | (18.9) | 18 | (56.3) | 0.049 |
| 20q13.2 | 7 | (18.9) | 18 | (56.3) | 0.049 |
| 20q13.33 | 7 | (18.9) | 18 | (56.3) | 0.049 |
| **Loss** |  |  |  |  |  |
| None |  |  |  |  |  |
| **CN-LOH** |  |  |  |  |  |
| None |  |  |  |  |  |

Supplementary Table 6: Comparison of gene mutation frequencies among IFN, LGD and HGD using NGS

|  | IFN (%) | LGD (%) | HGD (%) | *p*-value |
| --- | --- | --- | --- | --- |
| Total | 30 | 37 | 28 |  |
| *KRAS* | 2 (6.7) | 2 (5.1) | 3 (9.4) | 0.8854 |
| *TP53* | 2 (6.7) | 1 (2.6)* | 10 (31.3)* | 0.0009 |
| *APC* | 1 (3.3) | 6 (15.4) | 7 (21.9) | 0.0895 |
| *BUB1* | 1 (3.3) | 0 | 0 | 0.297 |
| *PIK3CA* | 1 (3.3) | 0 | 0 | 0.297 |
| *PTEN* | 1 (3.3) | 0 | 0 | 0.297 |
| *SMAD4* | 1 (3.3) | 0 | 0 | 0.297 |
| *TGFBR2* | 1 (3.3) | 0 | 0 | 0.297 |

IFN, intramucosal foveolar neoplasia; LGD, low-grade dysplasia;

HGD, high-grade dysplasia; NGS, next-generation sequencing

*, p < 0.01

Supplementary Table 7: Comparison of *APC* mutation frequency among IFN, LGD and HGD using NGS

| Gene | Codon | Exon | Type | Consequence | IFN (%) | LGD (%) | HGD (%) |
| --- | --- | --- | --- | --- | --- | --- | --- |
| Total mutations | |  |  |  | 1 | 6 | 7 |
| *APC* | R232* (Cga/Tga) | 7 | Transition | Nonsense | 0 | 0 | 1 (14.3) |
|  | R283* (Cga/Tga) | 9 | Transition | Nonsense | 0 | 0 | 2 (28.6) |
|  | Y500* (taT/taG) | 12 | Transversion | Nonsense | 0 | 1 (16.7) | 0 |
|  | R564* (Cga/Tga) | 14 | Transition | Nonsense | 0 | 0 | 1 (14.3) |
|  | S688* (tCa/tAa) | 16 | Transversion | Nonsense | 0 | 1 (16.7) | 0 |
|  | R876* (Cga/Tga) | 16 | Transition | Nonsense | 0 | 1 (16.7) | 0 |
|  | R1114* (Cga/Tga) | 16 | Transition | Nonsense | 0 | 0 | 1 (14.3) |
|  | R1450* (Cga/Tga) | 16 | Transition | Nonsense | 0 | 1 (16.7) | 0 |
|  | K1462X (aAG/a) | 16 | Deletion | Frameshift | 0 | 2 (33.3) | 0 |
|  | E1554EX (gaa/gAaa) | 16 | Insertion | Frameshift | 1 (100) | 0 | 1 (14.3) |
|  | E1573* (Gaa/Taa) | 16 | Transversion | Nonsense | 0 | 0 | 1 (14.3) |

IFN, intramucosal foveolar neoplasia; LGD, low-grade dysplasia; HGD, high-grade dysplasia; NGS, next-generation sequencing

Supplementary Table 8: Comparison of *APC* exon mutation and mutation types among IFN, LGD and HGD using NGS

|  |  | IFN (%) | LGD (%) | HGD (%) |
| --- | --- | --- | --- | --- |
| Total mutations |  | 1 | 6 | 7 |
| Exon | Exon 7 | 0 | 0 | 1 (14.3) |
|  | Exon 9 | 0 | 0 | 2 (28.6) |
|  | Exon 12 | 0 | 1 (16.7) | 0 |
|  | Exon 14 | 0 | 0 | 1 (14.3) |
|  | Exon 16 | 1 (100) | 5 (83.3) | 3 (42.9) |
|  |  |  |  |  |
| Type | Transition | 0 | 2 (33.3) | 5 (71.4) |
|  | Transversion | 0 | 1 (16.7) | 1 (14.3) |
|  | Deletion | 0 | 3 (50.0) | 0 |
|  | Insertion | 1 (100) | 0 | 1 (14.3) |
|  |  |  |  |  |
| Consequence | Missense | 0 | 0 | 0 |
|  | Nonsense | 0 | 4 (66.7) | 6 (85.7) |
|  | Frameshift | 1 (100) | 2 (33.3) | 1 (14.3) |

IFN, intramucosal foveolar neoplasia; LGD, low-grade dysplasia; HGD, high-grade dysplasia; NGS, next-generation sequencing

Supplementary Table 9: Comparison of *KRAS* mutation frequency among IFN, LGD and HGD using NGS

| Gene | Codon | Exon | Type | Consequence | IFN (%) | LGD (%) | HGD (%) |
| --- | --- | --- | --- | --- | --- | --- | --- |
| Total mutations | |  |  |  | 2 | 2 | 3 |
| *KRAS* | G12A (gGt/gCt) | 2 | Transversion | Missense | 0 | 1 (50.0) | 0 |
|  | G12C (Ggt/Tgt) | 2 | Transversion | Missense | 1 (50.0) | 0 | 1 (33.3) |
|  | G12D (gGt/gAt) | 2 | Transition | Missense | 0 | 0 | 2 (66.7) |
|  | G12S (Ggt/Agt) | 2 | Transition | Missense | 0 | 1 (50.0) | 0 |
|  | G13D (gGc/gAc) | 2 | Transition | Missense | 1 (50.0) | 0 | 0 |

IFN, intramucosal foveolar neoplasia; LGD, low-grade dysplasia; HGD, high-grade dysplasia; NGS, next-generation sequencing

Supplementary Table 10: Comparison of *KRAS* mutation frequency among IFN, LGD and HGD using NGS

| Gene |  | IFN (%) | LGD (%) | HGD (%) |
| --- | --- | --- | --- | --- |
| Total mutations | | 2 | 2 | 3 |
| Exon | Exon 2 | 2 (100) | 2 (100) | 3 (100) |
|  |  |  |  |  |
| Type | Transition | 1 (50.0) | 1 (50.0) | 2 (66.7) |
|  | Transversion | 1 (50.0) | 1 (50.0) | 1 (33.3) |
|  |  |  |  |  |
| Consequence | Missense | 2 (100) | 2 (100) | 3 (100) |
|  | Nonsense | 0 | 0 | 0 |
|  | Frameshift | 0 | 0 | 0 |

IFN, intramucosal foveolar neoplasia; LGD, low-grade dysplasia; HGD, high-grade dysplasia; NGS, next generation sequencing
